# *Aspalathus linearis* (Rooibos) Targets Adipocytes and Obesity-Associated Inflammation

**DOI:** 10.3390/nu15071751

**Published:** 2023-04-03

**Authors:** Rawan Nehme, Arthur Chervet, Caroline Decombat, Lucie Longechamp, Adrien Rossary, Rebecca Boutin, Amandine Rousset, François Senejoux, Caroline Vachias, Céline Auxenfans, Didier Fraisse, Jean-Baptiste Guyon, Edith Filaire, Jean-Yves Berthon, Mona Diab-Assaf, Laetitia Delort, Florence Caldefie-Chezet

**Affiliations:** 1 Université Clermont-Auvergne, INRAE, UNH, Unité de Nutrition Humaine, CRNH-Auvergne, 63000 Clermont-Ferrand, France; 2 Greentech, Biopôle Clermont-Limagne, 63360 Saint-Beauzire, France; 3 iGReD (Institute of Genetics, Reproduction and Development), Université Clermont Auvergne, UMR CNRS 6293-INSERM U1103, Faculté de Médecine, 28 Place Henri-Dunant, 63000 Clermont-Ferrand, France; 4 Banque de Tissus et de Cellules, Hôpital Edouard-Herriot, 69000 Lyon, France; 5 Equipe Tumorigénèse Pharmacologie Moléculaire et Anticancéreuse, Faculté des Sciences II, Université Libanaise Fanar, Beirut 1500, Lebanon

**Keywords:** Rooibos, *Aspalathus linearis*, obesity, inflammation, antioxidant, adiponectin, leptin, adipogenesis, macrophages

## Abstract

Excess weight and obesity are the fifth leading cause of death globally, and sustained efforts from health professionals and researchers are required to mitigate this pandemic-scale problem. Polyphenols and flavonoids found in *Aspalathus linearis*—a plant widely consumed as Rooibos tea—are increasingly being investigated for their positive effects on various health issues including inflammation. The aim of our study was to examine the effect of Rooibos extract on obesity and the associated low-grade chronic inflammatory state by testing antioxidant activity, cytokine secretions, macrophage polarization and the differentiation of human adipocytes through the development of adipospheroids. Rooibos extract significantly decreased ROS production and the secretion of pro-inflammatory cytokines (IFN-γ, IL-12, IL-2 and IL-17a) in human leukocytes. Additionally, Rooibos extract down-regulated LPS-induced macrophage M1 polarization, shown by a significant decrease in the expression of pro-inflammatory cytokines: *TNFα*, *IL-8*, *IL-6*, *IL-1β* and *CXCL10*. In addition, Rooibos inhibited intracellular lipid accumulation and reduced adipogenesis by decreasing the expression of *PPARγ*, *Ap2* and *HSL* in adipospheroids. A significant decrease in *leptin* expression was noted and this, more interestingly, was accompanied by a significant increase in *adiponectin* expression. Using a co-culture system between macrophages and adipocytes, Rooibos extract significantly decreased the expression of all studied pro-inflammatory cytokines and particularly *leptin*, and increased *adiponectin* expression. Thus, adding Rooibos tea to the daily diet is likely to prevent the development of obesity associated with chronic low-level inflammation.

## 1. Introduction

In 2022, the obesity pandemic was one of the most prominent health issues that occupied the attention of scientists, healthcare providers and the World Health Organization (WHO), who classified it as one of the ten biggest threats to human health [1]. Obesity has now surpassed categorization as a “medical condition”, and is considered a true chronic and metabolic disease with its own pathophysiology and rate of comorbidity [2]. It is estimated that by 2030, one billion people—that is, 1 in 5 women and 1 in 7 men—will be obese, according to the World Obesity Atlas 2022 [3]. Obesity is clinically defined as a body mass index (BMI) over 30 kg/m^2^, and is characterized by an abnormal increase in adipose tissue, leading to physiological dysfunction of the human organism [4]. It has a complex etiology that could include genetic and epigenetic factors, environmental factors such as overconsumption along with a sedentary lifestyle, and also psychological and endocrinological factors [5]. The expansion of adipose tissue is mainly due to adipogenesis, which involves the proliferation of preadipocytes and their differentiation into mature lipid-accumulating adipocytes [6]. The differentiation may be regulated by many effectors (including hormones and miRNAs), but the main regulators are transcription factors that inhibit or promote the expression of proteins required for adipogenesis, chief among them is Peroxisome proliferator-activated receptor gamma (PPARγ) [7].

In addition to the well-documented impact of obesity on numerous serious diseases, the COVID-19 pandemic has, once again, demonstrated the gravity of this health issue. In the last three years, more serious consequences from COVID-19, plus a higher rate of hospitalization and death, have been reported among the overweight and obese population than among the population within the normal weight range [8]. Obesity causes a series of metabolic dysregulations which could affect nearly every organ system, from the endocrine system to the cardiovascular system and the central nervous system [9]. It is associated with oxidative stress and chronic low-grade inflammation, characterized by the infiltration of an increasing number of immune cells—mainly macrophages—into the obese adipose tissue [10]. The resulting phenotypic switch in the adipose tissue leads to the appearance of inflamed dysfunctional adipocytes [11]. This event is recognized as the first stimulus for the development of obesity complications that include insulin resistance, diabetes, dyslipidemia, immune disorder, non-alcoholic fatty liver diseases, several cardiovascular issues (from hypertension to heart failure) and over 13 types of cancer [12,13]. The infiltration of immune cells into adipose tissue was first noticed in the 1960s and, in 2005, the accumulation of macrophages around dying adipocytes in a crown-like structure was shown. Since then, significant attention has been paid to this inflammatory reaction. The macrophages are classically activated by the adipose microenvironment into M1 macrophages, which are pro-inflammatory cells producing pro-inflammatory cytokines such as interleukin-1β (IL-1β), IL-6, IL-12 and TNFα [14]. The inflammation of the adipose tissue is persistent and fails to resolve, due to the continuous release of inflammatory mediators, including the cytokines previously mentioned, and adipokines (i.e., leptin) and the lack of secretion of anti-inflammatory adipokine (i.e., adiponectin) by adipocytes and resident immune cells [15].

It is indisputable that lifestyle modification is the ineluctable first step in managing obesity. This includes not only calorie restriction, but also several other new dietary interventions such as time-restricted eating, intermittent fasting and fasting-mimicking diets, along with engagement in physical activities. Unfortunately, such behavioral changes are insufficient in most cases and adjunctive treatment, including pharmacological therapies or surgery, is usually needed [16]. The main FDA-approved anti-obesity drugs are orlistat, phentermine/topiramate, naltrexone/bupropion, liraglutide [17] and, very recently, semaglutide [18]. However, most of these drugs are associated with increased adverse events, ranging from mild to potentially serious, which could lead to drug discontinuation [19,20]. Thus, there is an urgent need to find new and safer molecules that could treat obesity or prevent its development. Natural substances from various medicinal plants have shown a variety of pharmacological properties and served as sources of therapeutic drugs against various acute and chronic disorders [21]. Since several medicines originate from and/or are inspired by active ingredients in medicinal plants, the use of plant extracts and their isolated metabolites is gradually coming to be considered a necessity, and medicinal plants play a prominent role in the drug-discovery process [22].

Rooibos tea, produced from the south African legume *Aspalathus linearis* (Burm. f.) R. Dahlgren, is a well-known health-promoting drink that contains a unique blend of bio-active phytochemicals (flavonoids or plant-derived phenolic compounds or phenolic constituents), mainly aspalathin [23]. Rooibos tea has been consumed since the late 1700s, but its potent antioxidant activity and health-giving properties have only been reported in the past two decades, increasing its popularity [24]. Thus, Rooibos has attracted the attention of several research groups, and multiple in vivo and in vitro studies have demonstrated its significant effect on insulin resistance, diabetes, cardiovascular complications (mainly as an anti-hypertensive, through inhibition of angiotensin-converting enzyme), inflammation, viral infections, ovarian function, and others [24,25]. The anti-obesity potential of Rooibos extract was first reported in 2011 [26]; then, a research group demonstrated its effect on murine preadipocyte cells, but no more data were found on its impact on human adipocytes [27]. Thus, in this study, we further investigated the metabolic modulatory effect of Rooibos extract in an obesity concept, i.e., an obese inflamed adipose tissue microenvironment. We hypothesized that because of its antioxidant properties, Rooibos extract could interact with the inflammatory response of adipocytes. Ultimately, our study could unravel the cellular mechanisms by which Rooibos extract works, and highlight the use of this plant extract and/or its bioactives in the prevention and management of obesity associated with inflammation.

## 2. Materials and Methods

### 2.1. Chemicals and Reagents

All solvents (water, ethanol, acetonitrile) and chemicals (sodium acetate, acetic acid 100%, hydrochloric acid 37%, phosphoric acid, 2,4,6-Tris(2-pyridyl)-s-triazine (TPTZ), Trolox, DPPH (2,2-diphenyl1-pycrilhydrazyle), Ferric chloride hexahydrate, and Iron trichloride) were acquired from VWR (USA) at HPLC grade. Aspalathin standard (primary reference standard grade) was obtained from Merck (Darmstadt, Germany).

### 2.2. Preparation of Aspalathus linearis Extract

The aerial parts (leaves and twigs) of green Rooibos (*Aspalathus linearis*) were collected from the city of Malmesbury, South Africa. They were sun-dried and shredded into pieces between 0.1 and 0.5 mm. Then, 10 g of dried plant was extracted into a 200 mL ethanol–water mixture (50%, *v*/*v*) and stirred on a rotary shaker (at 120 rpm) for 4 h at room temperature. The mixture was then filtered with a filter paper in order to separate the solid and the extract. The extract was then concentrated using a rotary evaporator (at 40 °C) to obtain 1.2 g of dry powder. The powder was then dissolved in DMSO and stored at −20 °C. Final concentrations (0, 10, 25, 50 and 100 µg/mL) were obtained by diluting the extract in complete culture medium directly before use.

### 2.3. Quantification of Aspalathin Content by HPLC-UV

Aspalathin was analyzed by high-performance liquid chromatography (HPLC) linked to a 190–800 nm diode array detector (Agilent, Santa Clara, CA, USA). Analysis was performed with a Luna C18 (250 mm × 4.6 mm × 5 µm; Phenomenex) column, with a pre-column C18, at 20 °C and a flow rate of 1 mL/min. The initial mobile phase was composed of 85% solvent A (0.1% phosphoric acid in water) and 15% solvent B (acetonitrile), and those percentages were maintained for 25 min. The percentage of solvent B was then increased to reach 35% at 35 min and 90% at 40 min. This concentration was then held for 5 min, before following a linear gradient to return to 15% at 55 min. Then, 10 µL of a 5 mg/mL (water/methanol, 1/5, *v/v*) Rooibos extract solution was injected. Quantification was performed using UV at 288 nm with an aspalathin standard.

### 2.4. Determination of Antioxidant Activity by Ferric-Reducing Antioxidant Power (FRAP) Assay and DPPH Radical-Scavenging Activity

FRAP reagent solution was prepared by mixing acetate buffer (20 mM, pH 3.6), TPTZ solution (10 mM in 40 mM HCl) and FeCl_3_·6H_2_O solution (20 mM) in a 10:1:1 *v*/*v*/*v* ratio. Trolox dilution solutions were prepared (0.1 to 1 mg/L). Then, 50 μL of extract or Trolox standard solution, and 200 µL of FRAP reagent solution were mixed and incubated for one hour at 37 °C. Absorbance was measured at 593 nm. FRAP was calculated and expressed as µmol Trolox equivalent/g (dw).

DPPH reagent (0.180 g·L^−1^ in ethanol) was prepared. In test tubes, 100 μL samples of workable solutions (dilutions of dry extract in ethanol) were combined with 3.9 mL of DPPH reagent. The tubes were then kept in complete darkness for 30 min at room temperature. Absorbance was measured at 515 nm. The effective concentration value (EC_50_), which corresponds to the concentration of a sample producing a 50% reduction in the initial DPPH concentration, was determined.

### 2.5. Blood Cells, Cell Cultures and Reagents

#### 2.5.1. Blood Leukocyte Preparation

Blood was collected from healthy human volunteers (n = 3; Établissement Français du Sang, EFS, Clermont-Ferrand, France). Donors gave their written informed consent for the use of blood samples for research purposes under EFS contract no. 16-21-62 (in accordance with articles L1222-1, L1222-8, L1243-4 and R1243-61 of the French Public Health Code). Blood leukocytes were obtained by hemolytic shock using ammonium chloride solution (NH_4_Cl 155 μM; NaHCO_3_ 12 μM, EDTA 0.01 μM). Leukocytes were then centrifuged and suspended in Roswell Park Memorial Institute 1640 Medium (RPMI-1640, Gibco, ThermoFisher Scientific, Waltham, MA, USA) supplemented with fetal bovine serum (FBS, 10%) (Eurobio Scientific, Saclay, France), gentamicin (50 μg/mL) and glutamine (Gln, 2 mM) (ThermoFisher Scientific, USA).

#### 2.5.2. PBMC Preparation

Blood buffy coats were collected from healthy human volunteers (n = 3 volunteers) and carefully layered on a simple gradient of Ficoll–Histopaque 1077^®^ (Sigma-Aldrich, St. Louis, MO, USA). After centrifugation (400× *g*, 40 min at 20 °C), the first layer of plasma was aspirated, yielding a phase of monocytes and lymphocytes (peripheral blood mononuclear cells, PBMCs) just above the 1.077 g/mL layer. The phase with PBMCs was washed with RPMI and centrifuged (5 min, 400× *g*) twice, and then suspended in 5 mL of supplemented RPMI (FBS 10%, gentamicin 50 μg/mL, and Gln 2 mM). Cell preparation was adjusted to 10^6^ cells/mL for assays.

#### 2.5.3. Human Monocytic Leukemia Cells

THP-1 cells (American Type Culture Collection, TIB-202TM) were cultured in a RPMI-1640 medium supplemented with 10% FBS, 2 mM Gln and 50 µg/mL gentamicin. For differentiation, THP-1 cells (4 × 10^5^/mL) were incubated in 6-well plates in a complete growth medium containing 16.2 nM phorbol 12-myristate 13-acetate (PMA, Sigma-Aldrich) for three days. Macrophages were then polarized into M1-like macrophages by incubation with 20 ng/mL of IFN-γ (Gibco) and 10 pg/mL of lipopolysaccharides (LPS, Sigma) for 24 h.

#### 2.5.4. Adipose Cells

Two preadipocyte cell types were used in this study:

- Preadipocyte cells were obtained from patients undergoing surgery for cosmetic purposes without associated pathology in accordance with the Helsinki Declaration, from anonymous healthy donors. Surgical residue was harvested in accordance with French regulations, including a declaration to the Research Ministry (DC no. 2008162) and procurement of written informed consent from the patients. Four different strains were obtained from obese women (BMI > 30). For differentiation into mature adipocytes (Mas), cells were seeded at confluence (33,500 cells/cm^2^) in a differentiation medium consisting of Dulbecco’s modified Eagle medium (DMEM/F12 (1:1), Gibco) supplemented with 10% FBS, 1% Gln, hydrocortisone (25 mg/mL), insulin (3.5 mg/mL), T3 (6.5 mg/mL), dexamethazon (980 mg/mL), rosiglitazone (1.78 mg/mL), isobutyl-methylxanthine (IBMX) (100 mg/mL, only for the first 3 days), and gentamycin (50 mg/mL). The medium was replaced every two days. Mas were obtained after 8 days of differentiation.
- The human subcutaneous preadipocyte cells were purchased from Lonza Group Ltd. (Basel, Switzerland). The initiation and expansion of the cells used Preadipocyte Growth Medium-2 (Preadipocyte Basal Medium-2 supplemented with 10% FCS, 2 mM Gln, 30 µg/mL genistein and 15 ng/mL ampicillin). To induce differentiation, cells were plated at 10,000 cells/cm^2^ in Preadipocyte Growth Medium-2. After 24 h, the culture medium was changed to adipocyte differentiation medium, consisting of Preadipocyte Growth Medium-2 supplemented with insulin, dexamethasone, indomethacin and IBMX. Cells were allowed to differentiate for 8 days with no further medium change.

All the cells used were cultured in a 5% CO_2_-humidified incubator at 37 °C.

### 2.6. Kinetics of ROS Production by Blood Leukocytes

The blood leukocyte preparation was adjusted to 10^6^ cells/mL and placed in 96-well plates incubated with 0, 10, 25, 50 or 100 µg/mL Rooibos extract and dihydrorhodamine 123 (DHR 123, 1 μM, Sigma-Aldrich), and stimulated, or not (to check to the stimulation efficiency), by 1 µM PMA for 120 min, as previously described by Cholet et al. [28]. The fluorescence intensity of rhodamine 123, which is the product of DHR 123 oxidation by ROS, was recorded every 5 min for 120 min (excitation/emission: 485/538 nm) using a microplate fluorometric reader (Tecan Spark^®^, Männedorf, Switzerland). Results were presented as a percentage of ROS production of stimulated treated cells compared to the control, which corresponded to stimulated untreated cells (100%).

### 2.7. Leukocyte Viability

The same cell preparation adjusted to 10^6^ cells/mL with supplemented RPMI (FBS 10%, gentamicin 50 μg/mL and Gln 2 mM) was placed in 96-well polystyrene plates incubated with Rooibos extract at four different concentrations (10, 25, 50 or 100 µg/mL), PMA (0 or 1 µM), and resazurin (25 µg/mL) [28]. Fluorescence (excitation/emission: 544/590 nm) was recorded every 30 min for 2 h using the microplate fluorometric reader (Fluoroskan^®^, Ascent FL, ThermoFisher Scientific, USA).

### 2.8. Determination of Cytokine Concentrations

PBMCs (10^6^ cells/mL) (n = 3 volunteers) were incubated with or without phytohemagglutinin (PHA, 5 µg/mL, Sigma-Aldrich) and Rooibos extract (0 or 50 µg/mL) for 24 h. ProcartaPlex™ Immunoassays (ThermoFisher Scientific, USA) were used for all assays [28]. All samples were run in triplicate and were assayed for 10 human cytokines (IFN-γ, IL-12 p70, IL-1β, IL-2, IL-23, IL-6, IL-8, IL-17a, MIP-1α, and TNFα). Cytokine levels were measured using optimal concentrations of standards and antibodies in accordance with the manufacturer’s instructions. After completion of all the steps in the assay, the plates were read in the Luminex Bio-Plex 200 System (Biorad, France) and the data were analyzed using Bio-Plex Manager™ 4.1 software with five-parameter logistic regression (5PL) curve fitting.

### 2.9. Quantification of Lipid Accumulation

Oil Red O solution (0.5% in isopropanol) was purchased from Sigma, and staining was performed following the supplier’s recommendations. In brief, 3 parts Oil Red O solution were mixed with 2 parts water prior to the experiment to make up the working solution (0.2% in 60% isopropanol). Cells were washed twice with PBS before fixation with 4% paraformaldehyde (30 min at room temperature). The paraformaldehyde solution was then discarded, and cells were washed twice with water before being incubated with a 60% isopropanol solution for 5 min and with the working Oil Red O solution for 20 min. After 5 more washes with water, the cells were observed under the microscope. In order to quantify the staining, the fixed dye was redissolved in a fixed volume of 100% isopropanol, and the optical density was measured at 490 nm using the microplate fluorometric reader (Tecan Spark^®^).

### 2.10. Evaluation of Gene Expression by Quantitative Real-Time PCR (qRT-PCR)

Following the incubation, total RNA was extracted with TRIZOL reagent (Invitrogen, ThermoFisher Scientific). After the evaluation of the quantity and purity (Tecan Spark^®^), DNase treatment was applied to remove any remaining genomic DNA (DNase I Amplification grade, Invitrogen) and cDNA reverse transcription (HighCap cDNA RT Kit RNAse inhib, Invitrogen) was performed according to the manufacturer’s recommendations. The cDNA samples were diluted to 5 ng/μL. Amplification reaction assays were carried out using SYBRGreen PCR Master Mix (Applied Biosystems, USA) and primers (Table 1) on a StepOne^TM^ machine (Applied Biosystems). The thermal cycling conditions were 50 °C for 2 min followed by an initial denaturation step at 95 °C for 10 min, 40 cycles at 95 °C for 30 s, 60 °C for 30 s and 72 °C for 30 s. The experiments were carried out in duplicate for each data point. The reference gene β-actin was used as an internal control for the normalization of RNA quantity and quality differences among the samples. Genes were considered significantly expressed and their transcript measurable if their corresponding Ct value was less than 35. The relative quantification method (RQ = 2^–ΔΔCT^) was used to calculate the relative gene expression of given samples with ΔΔCT = [ΔCT (sample1) − ΔCT (sample2)] and ΔCT = [CT (target gene) − CT (reference gene)]. Paired t-tests were used for comparisons of gene-expression levels with at least two valid pairs of values. The rate of false discovery due to multiple testing was controlled by means of the Benjamini–Hochberg procedure for each comparison separately. Three independent experiments were performed.

### 2.11. Adipospheroid Generation

The objective was to generate 3D spheroids using human preadipocytes in order to mimic the three-dimensionality of adipose tissue with its histological and physiological properties. Two methods were tested in order to obtain the spheroids. We used two scaffold-free systems in which the cells cannot adhere to the support, and thus can multiply in 3D and form multicellular micro-tissues.

In the first method, 20 g/L agarose (ThermoFisher Scientific) of sterilized 0.9% *w/v* NaCl (Sigma-Aldrich) was prepared, sterilized for 20 min at 120 °C and put in MicroTissues^®^ 3D Petri Dishes^®^ (81 wells, Sigma-Aldrich) according to the manufacturer’s instructions to obtain an agarose mold [29]. In these molds, cell adhesion is inhibited, and cells present within the suspension spontaneously agglomerate to form spheroids by promoting intercellular adhesion molecules. Next, a total of 200,000 preadipocyte cells/agarose mold were seeded in the agarose mold and cultured in DMEM/F12 medium (supplemented with 10% FBS, 1% Gln) at 37 °C in 5% CO_2_, resulting in the assembly of 81 potential adipospheroids per agarose mold. At day 2, the differentiation medium was added as described previously.

The second method consists of using 96-well ultra-low-binding U-shaped-bottom plates (Corning, Somerville, MA, USA) that promote cell aggregation and spheroid formation. A total of 10,000 preadipocytes/well were seeded and cultured in the DMEM/F12 medium (supplemented with 10% FBS and 1% Gln); then, the spheroids were differentiated for 8 days, with the medium being changed every two days. Imaging of spheroids in these low-binding U-shaped-bottom plates was performed using Incucyte^®^ (Sartorius, Göttingen, Germany), an image acquisition and analysis system with a 10× objective. The total spheroid area (diameter) was measured using Fiji and Incucyte^®^ software. A minimum of three spheroids were measured for each time point.

### 2.12. Confocal Microscopy

To assess the differentiation of adipocytes in the adipospheroids, we observed and analyzed the lipid content by confocal microscopy. Firstly, adipospheroids were washed in Dulbecco’s phosphate-buffered saline (DPBS, Gibco) after harvesting and subsequently fixed for 1 h at room temperature in a formalin solution (neutral buffered 10%, Sigma-Aldrich). The adipospheroids were then stained overnight, protected from light, in 1:2000 Bodipy-FL (1 mg/mL, Invitrogen), 1:500 Dapi (1 mg/mL) and 1:500 Triton X-100 (5 mg/mL) (ThermoFisher Scientific). After staining, the adipospheroids were washed three times for 5 min in DPBS and then mounted in a special support (ISO18 10 mm deep, Ispacer^®^, SUNJin Lab) using Vectashield (H1000 Vector, Eurobio Scientific) as a mounting medium to observe the entire adipospheroids in 3D and avoid cutting or crushing them. The stained adipospheroids were analyzed by confocal microscopy using a Micro Zeiss Cell Observer Spinning Disk with Yokogawa CSU-1X scanning unit (×20 Plan Apochromat 20×/0.8 M27). Bodipy FL was excited using a 488 nm laser paired with a 509/22 nm bandpass filter. DAPI was excited using a 405 nm laser paired with a 450/50 nm bandpass filter. Equal light settings were used for all images within an experiment and sub-figure.

### 2.13. Co-Culture between Macrophages and Adipospheroids/Evaluation of Cell–Cell Interactions

A co-culture system was used between THP-1 cells seeded at the bottom of wells and adipospheroids added in inserts, allowing the effect of Rooibos extract on the interaction between the two cell types through a porous membrane (Transwell culture system, porosity 0.4 µm) to be assessed. THP-1 were seeded at the bottom of wells (450,000 cells/cm^2^) and the differentiation into adherent macrophages was induced by adding PMA for 3 days. Next, the macrophages were co-cultured with the mature adipospheroids, with or without the presence of IFN-γ, LPS and Rooibos extract (50 µg/mL). After 24 h of incubation, total RNA was extracted with TRIZOL, and qRT-PCR was performed as described previously. The experiments were carried out at least three times.

### 2.14. Statistical Analysis

All the experiments were performed 3–6 times; data are presented as mean ± SD. The data were analyzed using one-way ANOVA or linear-regression analysis in GraphPad Prism software version 8 (GraphPad Software, San Diego, USA). Statistical significance among several groups was assessed using one-way ANOVA followed by Dunnett’s *t*-test. Statistical significance between the two groups was evaluated by Student’s t-test. The *p* values were determined, and values < 0.05, < 0.01, < 0.001, 0.0001 (*, **, ***, ****, respectively) were considered significant.

## 3. Results

### 3.1. Antioxidant and Anti-Inflammatory Impact of Rooibos Extract

#### 3.1.1. Aspalathin Content and Antioxidant Capacity

The analysis of the main compound in the extract showed the presence of 7.95% of Aspalathin (dihydrochalcone *C*-glycoside) in the dry extract (Aspalathin content (AC) ≈ 80 g·kg^−1^).

**Figure 1:**
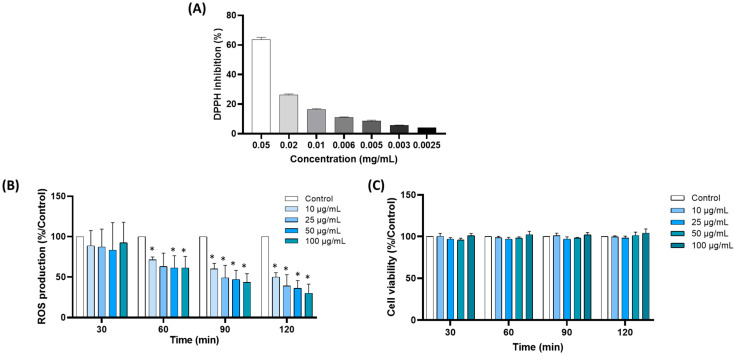
Antioxidant activity of Rooibos extract. (**A**) Free-radical scavenging activity toward DPPH radical; (**B**) production of ROS by blood leukocytes with different concentrations of Rooibos. Cells were incubated with Rooibos extract (0, 10, 25, 50 and 100 µg/mL) and stimulated with PMA (1 µM). Data shown as mean ± SD; (**C**) effect of Rooibos extract on leukocyte viability. Cells were treated with the indicated concentrations of Rooibos for 2 h, and then cell viability was measured. Data shown as mean ± SD (control corresponded to stimulated untreated cells = 100%); * *p* < 0.05 compared with control.

The radical-scavenging capacity of the extract was determined using DPPH and FRAP assays. The following FRAP and DPPH values, reflecting the direct antioxidant activity of the extract, were found for the FRAP assay; 4543 ± 143 µmol Trolox equivalent was found in 1 g of dry extract. For the free-radical scavenging activity toward the DPPH radical, the effective concentration value (EC_50_), determined by a 50% reduction in the initial DPPH concentration, was 0.00895 mg (of dry extract)/mL (Figure 1A). Thus, high direct scavenging activity was found in the Rooibos extract.

#### 3.1.2. Rooibos Extract Inhibited ROS Production by Blood Leukocytes

To investigate the potential antioxidant effects of Rooibos extract, we examined its effect on ROS production by blood leukocytes triggered by PMA. PMA stimulation resulted in a significant increase in ROS production after 1 h of incubation. Incubation with Rooibos extract inhibited the production of ROS in a dose-dependent manner (Figure 1B). The decrease was significant after 1 h at 10 µg/mL (≈30%). After 2 h, a significant reduction of minimum 50% was obtained, with a reduction of more than 60% at 50 µg/mL and above (−50%, −61%, –64% and –70% for 10, 25, 50 and 100 µg/mL, respectively). This effect was not the consequence of a decrease in cell viability or proliferation, as the viability assay did not show any significant difference between cells incubated with and without Rooibos extract in the concentration range 10–100 µg/mL after 2 h (Figure 1C). In light of these results, the concentration of 50 µg/mL was selected for further investigations.

#### 3.1.3. Rooibos Impacted PBMC Cytokine Secretion

**Figure 2:**
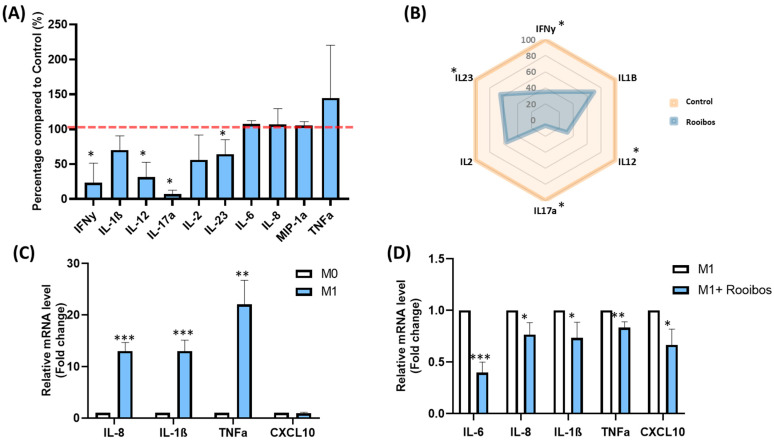
Impact of Rooibos extract on cytokines. (**A**) Measurement of ten pro-inflammatory cytokines secreted by PBMCs using Procarta-Plex™ Immunoassays. Cells were incubated with Rooibos extract (50 µg/mL) and stimulated with PHA (5 µg/mL) for 24 h. Data shown as mean ± SD; (**B**) summary of the overall effect of Rooibos extract on the main pro-inflammatory cytokines in PHA-stimulated PBMCs; (**C**) THP-1 macrophages were stimulated with 20 ng/mL IFN-γ  +  100 ng/mL LPS as described in the Materials and Methods section, and the mRNA expression of *IL-6*, *TNFα*, *IL-1β*, *IL-8* and *CXCL10* was measured by qRT-PCR. *β-Actin* was used as an internal control. M0 macrophages were used as a control; (**D**) the mRNA expression of *IL-6*, *TNF-α*, *IL-1β*, *IL-8* and *CXCL10* was measured by qRT-PCR in cells treated or untreated with 50 μg/mL Rooibos extract, and stimulated with LPS/IFN-γ. *β-Actin* was used as an internal control. Untreated M1 macrophages were used as a control. All data represent the means of 3–5 replicates ±  SD. * *p* < 0.05, ** *p* ≤ 0.01, *** *p* ≤ 0.001 represent significant differences compared with the control.

In order to determine the effect of Rooibos extract on inflammatory response, total PBMCs were stimulated by PHA in the presence or absence of 50 µg/mL of Rooibos extract, and the concentration of ten cytokines secreted in the supernatant was measured with ProcartaPlex™ Immunoassays. Rooibos extract significantly decreased the production of four pro-inflammatory cytokines, reflecting its anti-inflammatory effect (Figure 2A). A hugely significant decrease in IL-17a secretion(+ 90%, *p* < 0.05), followed by decreases in IFN-γ (–76%, *p* < 0.05), IL-12 (–60%, *p* < 0.05) and IL-23 (–46%, *p* < 0.05) were observed in stimulated PBMCs cultured with Rooibos extract in comparison to untreated PBMCs. IL-2 concentration was decreased by almost 45% (ns). Despite a downward trend, Rooibos extract was not able to significantly impact IL-1β secretion (−30% vs. untreated cells) due to interindividual variations. On the other hand, the secretion of IL-6, IL-8 and MIP-1α was not affected in stimulated treated cells vs. untreated cells, and a non-significant increase in TNFα secretion was observed, mainly due to high variations between donors. Figure 2B is a summary of the overall effect of Rooibos on the main pro-inflammatory cytokines in PHA-stimulated PBMCs. It can be observed that Rooibos extract modified the behavior of the pro-inflammatory cytokines.

#### 3.1.4. Rooibos Modulated the Polarization of Macrophages toward M1-Type

To better investigate the anti-inflammatory effect of Rooibos extract, the production of pro-inflammatory cytokines was quantified in M1 inflammatory macrophages in the presence or absence of 50 µg/mL Rooibos extract. For this purpose, THP-1 cell lines were activated and transformed into M0, and then polarized into M1-type macrophages by adding IFN-γ and LPS. M1 macrophages showed cellular elongation. As expected, the mRNA expression of *IL-1β*, *TNFα*, *IL-6*, *IL-8* and *CXCL10* (M1 macrophage markers) was significantly upregulated in the M1 population (Figure 2C), validating the successful polarization of monocytes into M1-polarized macrophages. Interestingly, Rooibos extract was able to attenuate the macrophage response to M1 pro-inflammatory activation by significantly reducing all pro-inflammatory cytokines (Figure 2D).

### 3.2. Impact of Rooibos Extract on Inflammatory State of Adipose Tissue

#### 3.2.1. Rooibos Extract Reduced Lipid Accumulation in Differentiated Human Adipocytes

**Figure 3:**
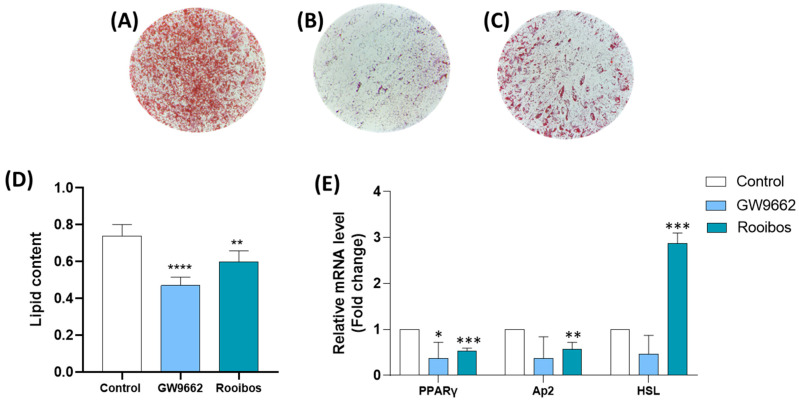
Reduction in intracellular triglyceride content and the expression of adipocyte-related genes in differentiated human adipocytes as a result of Rooibos extract; (**A**) differentiated human adipocyte controls cultured without Rooibos extract and GW9662; (**B**) differentiated adipocytes in the presence of GW9662; (**C**) differentiated adipocytes in the presence of 50 µg/mL Rooibos extract; (**D**) quantification of Oil Red O staining intensity measured (at 490 nm) after 8 days of chronic exposure to 50 µg/mL of Rooibos extract or GW9662. Results for the Oil Red O staining shown are from a representative experiment which was repeated two more times in quadruplicate with similar trends; (**E**) differentiating human adipocytes were grown in the presence and absence of 50 µg/mL of Rooibos extract until day 8. Total RNA isolated from untreated cells was used as the control. Data shown are the relative mRNA expression of *PPARγ*, *Ap2*, and *HSL*, normalized to *β-actin*. Significance is indicated as * where *p* ≤ 0.05, ** where *p* ≤ 0.01, *** where *p* ≤ 0.001 and **** where *p* ≤ 0.0001 and n = 3–6.

Next, we investigated the potential effect of Rooibos extract on obesity by determining its impact on adipogenesis. Microscopic analysis of differentiated Oil Red O-stained human adipocytes, cultured in the absence (Figure 3A) and presence of GW9662 which is a PPARγ agonist (Figure 3B) or Rooibos (Figure 3C), revealed the accumulation of intracellular lipid droplets. Chronic treatment with Rooibos extract appeared to inhibit lipid accumulation. Quantification of the extracted Oil Red O stain showed that the lipid content was significantly reduced by ∼36% (*p* ≤ 0.0001) when treated with GW9662 (used as a negative control) and ∼20% (*p* ≤ 0.001) with Rooibos as compared to the control (Figure 2D), indicating a reduction in intracellular lipid accumulation.

#### 3.2.2. Rooibos Extract Suppressed Adipocyte-Related Gene Expression during Differentiation

Differentiation of human adipocytes with 50 µg/mL of the Rooibos extract decreased the gene expression of *PPARγ* (Figure 3E) by 47% (*p* ≤ 0.001) and that of *Ap2* by 43% (*p* < 0.01) compared to untreated controls. Additionally, Rooibos extract significantly increased the expression of the *Lipase E*, *hormone-sensitive type (HSL*) gene by 186% (*p* ≤ 0.001).

#### 3.2.3. Rooibos Extract Affected Adipokine Secretion in Differentiated Human Adipocytes

**Figure 4:**
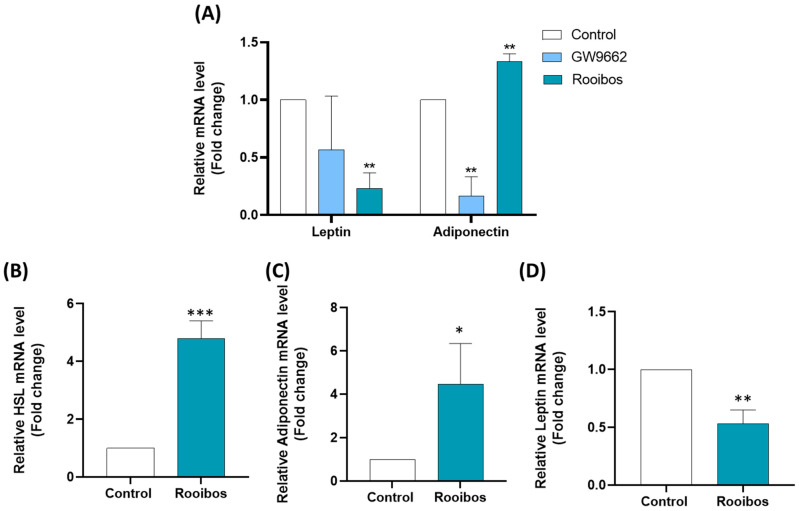
Adipokine expression in human adipocytes is affected by Rooibos treatment. Cells were exposed to 50 μg/mL of Rooibos extract for the full differentiation period. Total RNA isolated from untreated cells was used as the control. Data shown are the relative mRNA expression of *leptin* and *adiponectin* (**A**), normalized to *β-actin*. Cells were differentiated normally, and mature adipocytes were treated with 50 μg/mL Rooibos for 24 h. Total RNA isolated from untreated cells was used as the control. Data shown are the relative mRNA expression of *HSL* (**B**), *adiponectin* (**C**) and *leptin* (**D**), normalized to *β-actin*. Significance is indicated as * where *p* ≤ 0.05, ** where *p* ≤ 0.01 and *** where *p* ≤ 0.001 and n = 3.

In light of the importance of adipokines in obesity and related metabolic syndromes, it was important to study the effect of Rooibos extract on their expression. A substantial decrease of 80% (*p* ≤ 0.001) was observed in leptin gene (*LEP*) expression by differentiated human adipocytes after chronic treatment with Rooibos extract at a concentration of 50 µg/mL. Interestingly, adiponectin gene (*AdipoQ*) expression was significantly increased by 33% by this treatment condition (Figure 4A).

#### 3.2.4. Rooibos Affected Adipokine and HSL Secretion in Mature Adipocytes

Next, the impact of Rooibos extract was evaluated in human mature adipocytes. For that purpose, human preadipocytes were differentiated as previously described for 8 days. Next, the mature adipocytes were treated with 50 μg/mL Rooibos extract for 24 h and the expression of adipokines along with *HSL* was evaluated. Rooibos extract significantly increased the expression of *AdipoQ* by 346% and *HSL* by 380% compared to untreated controls (Figure 4B,C). A substantial decrease of 46% (*p* ≤ 0.001) was observed in *LEP* expression by mature human adipocytes after 24-hour treatment with 50 µg/mL of Rooibos extract (Figure 4D).

#### 3.2.5. Rooibos Extract Effect on Adipospheroids in the Presence or Absence of Inflammatory Macrophages

**Figure 5:**
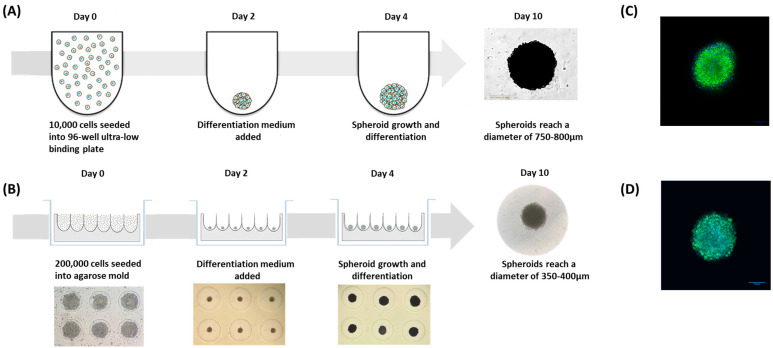
Generation of the 3D adipospheroids. (**A**) Using 96-well ultra-low-binding, U-shaped-bottom plates at 10,000 cells/well; (**B**) using an agarose mold with 81 wells at 200,000 cells/mold. With both methods, differentiation started at day 2 and ended at day 10; (**C**) confocal microscopy of adipospheroids formed in the plates with Bodipy and DAPI labeling in Vectashield mounting medium; (**D**) confocal microscopy of adipospheroids formed in the agarose mold with Bodipy and DAPI labeling in Vectashield mounting medium (Micro Zeiss Cell Observer Spinning Disk, ×20 Plan Apochromat 20×/0.8 M27).

To better demonstrate the potential anti-obesity effect of Rooibos, it was interesting to study its impact on adipose tissue mimetic by developing a 3D system that would allow cell expansion and more closely resemble in vivo conditions. The 3D cultures were shown to be biologically active, and can be maintained in long-term cultures and better reproduce adipose tissue’s morphological and physiological behaviors compared to their 2D counterparts [30]. To achieve this objective, we took advantage of the ability of human preadipocytes to agglomerate and form multicellular micro-tissues or “spheroids” when forced together on non-adherent surfaces. To set up the system, we used the same human subcutaneous preadipocytes whose ability to differentiate in vitro was demonstrated previously. Two techniques were employed to form the adipospheroids:

- The first method consisted of simply seeding the preadipocyte cells in specific U-shaped ultra-low-binding plates where cell adhesion is almost impossible (Figure 5A).
- The second method consisted of using agarose molds prepared with MicroTissues^®^ 3D Petri Dishes^®^ (Figure 5B). In these molds, 200,000 preadipocyte cells were seeded. After 24 h of incubation, cells present within the suspension spontaneously agglomerated to form loosely adhesive cell spheroids by promoting intercellular adhesion molecules, resulting in the assembly of 81 potential adipospheroids per agarose mold.

We then added the differentiating medium at day 2 and cells were allowed to continue differentiating until day 10 in both methods. To verify the differentiation of spheroids and their ability to mimic adipose tissue, the presence of lipid droplets was assessed by adding Bodipy, a green fluorescent dye with hydrophobic properties, ideal for staining lipids, and then analyzing the samples by confocal microscopy. In Figure 5C,D, the stained lipid droplets were clearly shown, indicating the total differentiation of adipospheroids in both methods.

**Figure 6:**
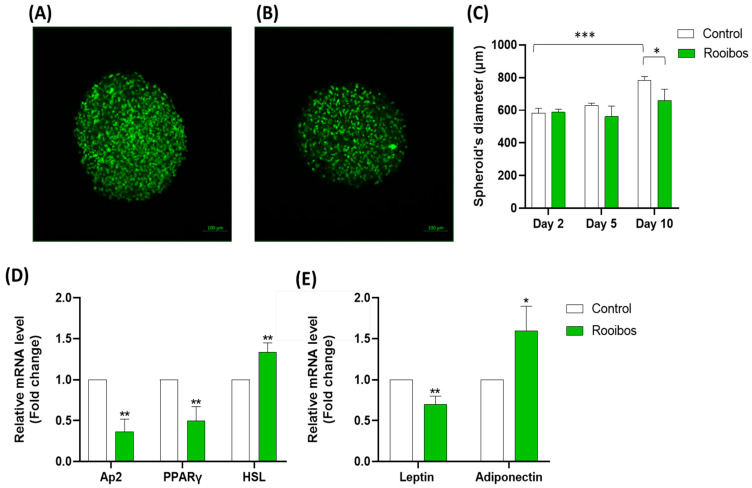
Confocal microscopy of adipospheroids stained with Bodipy labeling in Vectashield mounting medium formed in the absence (**A**) or presence (**B**) of 50 μg/mL Rooibos extract (Micro Zeiss Cell Observer Spinning Disk, ×20 Plan Apochromat 20×/0.8 M27). (**C**) Measurement of spheroids’ diameter using Fiji at days 2, 5 and 10 in the presence or absence of 50 µg/mL Rooibos extract. Differentiated human adipospheroids were grown in the presence or absence of 50 µg/mL of Rooibos extract until day 8. The mRNA expression of *Ap2, PPARγ* and *HSL* (**D**), and the adipokines (*leptin* and *adiponectin*) (**E**) was measured by qRT-PCR in these adipospheroids. Total RNA isolated from untreated adipospheroids was used as the control. Data shown are the relative mRNA expression normalized to β-actin. Significance is indicated as * where *p* ≤ 0.05, ** where *p* ≤ 0.01 and *** where *p* ≤ 0.001, and n = 3–6.

After verifying the models and the ability of adipospheroids to differentiate, we then examined whether Rooibos extract could regulate their differentiation. To test this, adipospheroids were formed using the ultra-low-binding plates, allowing the use of Incucyte^®^ to monitor the kinetics of the formation of adipospheroids and measure their diameters. The cells were differentiated in the presence or absence of 50 µg/mL of Rooibos extract from day 2. At day 10, the lipid droplets in the adipospheroids were stained using Bodipy and analyzed by confocal microscopy. Figure 6 shows the stained adipospheroids in the absence (Figure 6A) or presence (Figure 6B) of Rooibos extract. A decrease in the fluorescence in the adipospheroids treated with Rooibos extract was clearly observed under the confocal microscope (quantification of spheroid volume). The measurement of spheroid diameter using Fiji demonstrated that untreated adipospheroids expanded significantly more than the treated ones (Figure 6C). Similar to the adipose tissue that accumulates lipids when preadipocytes mature into adipocytes, our results indicated that, during differentiation, the spheroid volume increased over time, and as expected, Rooibos extract had the ability to inhibit this process. At the end of the differentiation period, we measured the expression of adipogenesis-related genes along with adipokines. The results indicated that the treated adipospheroids expressed less *Ap2*, *PPARγ* and *Leptin* (–63%, –50% and –30%, respectively) and more *adiponectin* and *HSL* (+60% and +33%, respectively) compared to untreated adipospheroids (Figure 6D,E). These results generally confirmed our results in 2D.

Next, we evaluated the effects of Rooibos extract on the interaction between the two inflammatory factors: adipocytes and macrophages.

**Figure 7:**
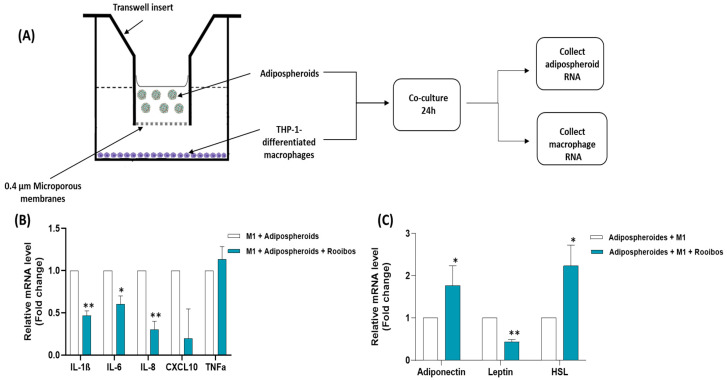
Effect of Rooibos on Adipocyte/Macrophage Interaction. (**A**) Diagram showing co-culture system where THP-1 were cultured in the lower chamber and adipospheroids were added after differentiation on a transwell insert; (**B**) the mRNA expression of IL-*6*, *TNFα*, *IL-1β*, *IL-8* and *CXCL10* was measured by qRT-PCR in M1 cells after 24 h of co-culture; (**C**) the mRNA expression of *adiponectin*, *leptin* and *HSL* was measured by qRT-PCR in the adipospheroids after 24 h of co-culture. Total RNA isolated from untreated M1 macrophages or adipospheroids was used as the control. Data shown are the relative mRNA expression of the mentioned genes normalized to *β-actin*, * where *p* ≤ 0.05 and ** where *p* ≤ 0.01 and n = 3.

We utilized a co-culture system to examine the impact of Rooibos extract on the inflammatory state caused by the interaction between M1 macrophages and adipocytes. THP-1 monocytic cells were seeded at the bottom of wells and differentiated into M0 by adding PMA. In parallel, adipospheroids were formed and differentiated in agarose molds as described previously. This method was selected for the co-culture since it generates a higher number of adipospheroids with 200,000 cells (81 vs. 20 spheroids for the low-binding plates method). The adipospheroids obtained were subsequently co-cultured with macrophages for 24 h in the presence or absence of IFN-γ, LPS to induce a pro-inflammatory profile, and 50 µg/mL of Rooibos extract. Next, total mRNA was extracted separately from the macrophages and adipospheroids to perform qPCR (Figure 7A). Samples were analyzed for the expression of markers of an M1-type phenotype, which represents a pro-inflammatory immune response. Five established M1 phenotype markers were selected: *IL-6*, *IL-8*, *IL-1β*, *TNFα* and *CXCL10*. In addition, three adipocytes’ markers were examined: *adiponectin*, *HSL* and *leptin*. The data showed that Rooibos extract generally decreased the inflammatory state, reflected by a significantly decreased expression of pro-inflammatory cytokines *IL-6*, *IL-8* and *IL-1β* in THP-1-differentiated macrophages by 60%, 70% and 53%, respectively, and the pro-inflammatory adipokine, *leptin*, by 56% in adipospheroids. These results were accompanied by a significant increase in the expression of *HSL* by 120% and the anti-inflammatory adipokine, i.e., *adiponectin*, by 70% in the adipospheroids compared to untreated co-culture (Figure 7B,C).

## 4. Discussion

Despite the fact that obesity is classified as a public health priority and is receiving special attention from the WHO and governments in all countries, its prevalence is still increasing worldwide, leading to great economic and social hardships along with high levels of stress on the health services in all countries. All of this goes to show that the obesity pandemic presents a real challenge for researchers, and new and improved ways to manage weight gain and prevent obesity are urgently needed.

Knowing that the adipose tissue is the most important organ to be targeted and studied when managing obesity, it is critically important to have a treatment that mainly targets adipocytes and adipose tissue. It is surprising that most of the available approved anti-obesity drugs mainly target the nervous system, mostly to decrease appetite and food consumption [18], instead of adipose tissue itself. In our study, we focused on the main factors in obesity responsible for chronic inflammation: adipocytes and macrophages. We investigated the effect of Rooibos extract on both cell types with a view to managing the obesity-associated chronic inflammation that leads to numerous serious diseases. Accordingly, in addition to the conventional 2D cultures, we used a 3D system, and we verified that the two tested techniques are simple and easily applicable culture protocols that allow the culturing and differentiation of human adipocytes with in vivo-like morphology in order to test new anti-obesity drugs. The two techniques allow the formation of mature adipospheroids where the preadipocytes can accumulate lipid droplets and gradually acquire an adipocyte-specific gene expression pattern.

Rooibos is known to contain high levels of valuable bioactive compounds (flavonoid antioxidants). Aspalathin is the specific flavonoid to Rooibos, and it is abundantly present in *Aspalathus linearis*, which explains the huge amount of interest in the potential health benefits of this flavonoid. The other four main flavonoids (nothofagin, orientin, isoorientin and vitexin) in Rooibos extract are not specific and may occur in other medicinal plants such as Leandra dasytricha [25]. The Rooibos extract used in our study contains 80 g·kg^−1^ aspalathin, which is in the range of the natural products containing Rooibos (8–12%) [31,32]. Generally, the consumption of 200 mL of Rooibos infusion would contribute 1.2–36 mg of aspalathin to the diet [25], depending on whether the product is fermented or green. For aspalathin and other bioactive compounds to be bio-effective, they must be bioavailable and reach their site of action. The various factors affecting bioavailability (absorption, distribution, metabolism and excretion) could lead to disparity in efficacy results between in vitro and in vivo studies. In this context, Huang et al. showed in their study that the absorption of aspalathin in a Caco-2 monolayer cell model was concentration dependent and could reach almost 100% of the initial dose in high concentrations [33]. The oral bioavailability of aspalathin from Rooibos extract or infusion has been evaluated in vivo using pig, mouse and vervet monkey models, in addition to three human studies. Overall, the studies have shown evidence of phase-II metabolites in blood circulation. The gluconidated, sulfated and methylated forms of aspalathin were found in the urine following phase-II liver metabolism after ingestion of Rooibos beverage or green Rooibos extract. The presence of aspalathin in the plasma was detected in one human study only, where the subjects drank 500 mL of green rooibos infusion, containing 287 mg aspalathin [34]. In the other two studies, no flavonoid metabolites were detected in the plasma and the authors noted that a strong affinity of the compounds for plasma carrier proteins such as serum albumin could be an explanation for undetectable levels in plasma [35,36]. On the one hand, we confirmed the potent antioxidant activity of Rooibos extract associated in our study with anti-inflammatory activity on blood leukocytes and THP-1-differentiated macrophages. In our study, macrophages polarized to M1-type by LPS/IFN-γ treatment showed a reduced pro-inflammatory response. The inhibition of pro-inflammatory cytokines involved in the inflammatory process, including IL-6, IL-8, IL-1β and TNFα, is one of the ways through which Rooibos extract seems to exhibit its anti-inflammatory activity. This effect could be due to the inhibitory effect of Rooibos on the NF-κB pathway, a mechanism demonstrated recently by Gbuza et al. which needs to be better elucidated [32]. The anti-inflammatory effect of our extract can be attributed to several active ingredients that, in addition to aspalathin, include vitexin, an apigenin-8-C-glucoside with various health benefits including antioxidation, anti-inflammation, fat reduction and glucose metabolism [37]. Thus, we confirm that this plant holds interest for the treatment of obesity-associated inflammation.

On the other hand, we showed that Rooibos decreased adipogenesis, decreasing the lipid accumulation process in the adipose tissue which is the major lipid storage site. This was mainly promoted by a significant decrease in the expression of *PPARγ*, the chief regulator of preadipocyte differentiation [38]. Interestingly, we also showed that Rooibos significantly increased the expression of HSL, which is an enzyme responsible for the breakdown of triacylglycerols into fatty acids in adipose tissue [39]. This could have a huge beneficial effect on weight loss because HSL is known to have a major impact on appetite, food intake and body weight [40]. Furthermore, it is essential to mention that the adipocytes are known to secrete over 100 cytokines involved in crosstalk with other cells. Among these, leptin and adiponectin have been shown to be therapeutically beneficial as a treatment for several obesity-related diseases [41]. The couple leptin/adiponectin is being extensively studied, due to the opposing effects of these two adipokines [42]. In obesity and associated metabolic diseases, a decreased plasma concentration of adiponectin along with increased leptin levels have been found. An imbalance between the pro-inflammatory leptin and the anti-inflammatory adiponectin has been shown to be associated with an elevated risk of developing obesity-related complications. Our findings showed that Rooibos extract significantly reduced *leptin* expression in mature adipocytes, once again reflecting its anti-inflammatory properties. Additionally, there is huge interest in adiponectin and its analogs as a potential therapy, not only for all metabolic syndromes but also for several cancer types [43]. To our knowledge, our team is the first to reveal that Rooibos significantly increases adiponectin expression in 2D and 3D human primary adipocyte cultures, contrary to the published results on 3T3-L1 murine adipocytes, where adiponectin secretion remained unchanged after Rooibos treatment [27]. To our knowledge, we are the first team to demonstrate this effect on human adipocytes, which could be attributed, once again, to aspalathin, in accordance with an in vivo study conducted on obese diabetic ob/ob mice, where the authors demonstrated that serum adiponectin levels were significantly increased by the dietary feeding of aspalathin [44]. In the longer term, it would be interesting to better investigate this impact by studying the effect of Rooibos on the AMPK signaling pathway, which is the main pathway activated by adiponectin [45], and the expression of adiponectin receptors in human adipocytes. Our results on adipocytes highlight the interest in Rooibos as a treatment for obese patients.

Finally, one of the major findings of our study was that Rooibos positively impacted the interaction between adipocytes and macrophages, mainly by decreasing the inflammatory state. The substantial weakness of in vitro experimentation is that it might not actually replicate the results in an organism, and one of the limitations of our study is the difficulty in reflecting the results in vivo. However, in our study, we used the 3D cell culture system, which is more physiologically relevant and predictive and responds better than the conventional 2D culture. In our co-culture system, we tried to mimic the reality of the adipose tissue and to copy the existing cell–cell interaction to better understand the mechanics of obese inflamed adipose tissue. Although not impossible, it is technically challenging to discover the exact concentration and form of Rooibos that could generate the same benefits in vivo.

Our results clearly suggest Rooibos as an effective and safe agent for obesity management and obesity development prevention in order to reduce the prevalence of obesity and its harmful consequences. To our knowledge, there have been no previously published studies where the effect of Rooibos on polarized macrophages has been evaluated. Furthermore, an obese inflamed adipose tissue microenvironment was created by culturing the adipospheroids (which are biologically more active than monolayer cultures) with M1 macrophages. This is the first time that Rooibos has been evaluated from the point of view of “obesity”. Nevertheless, a huge amount of work remains to be completed, which includes optimization of the dose, means of administration and the safety profile of concentrated aspalathin extracts. As a conclusion, our study verified that Rooibos extract could be a good candidate to promote healthy and well-functioning adipose tissue, which is important for whole-body metabolism and wellbeing, by reducing the low-grade inflammation observed in obesity.

## Figures and Tables

**Table 1:** PCR primer sequences.

Gene	Species	Forward Primer Sequence (5′-3′)	Reverse Primer Sequence (5′-3′)
*β-actin*	Human	CCTGGCACCCAGCACAAT	GCCGATCCACACGGAGTACT
*IL-8*	Human	CTGGCCGTGGCTCTCTTG	CCTTGGCAAAACTGCACCTT
*IL-1β*	Human	CCTGTCCTGCGTGTTGAAAGA	GGGAACTGGGCAGACTCAAA
*IL-6*	Human	GCTGCAGGCACAGAACCA	ACTCCTTAAAGCTGCGCAGAA
*TNFα*	Human	TCTTCTCGAACCCCGAGTGA	GGAGCTGCCCCTCAGCTT
*CXCL10*	Human	GGAAATCGTGCGTGACATTA	AGGAAGGAAGGCTGGAAGAG
*PPARγ*	Human	GGATTCAGCTGGTCGATATCAC	GTTTCAGAAATGCCTTGCAGT
*Ap2*	Human	ATCACATCCCCATTCACACT	ACTTGTCTCCAGTGAAAACTTTG
*HSL*	Human	GCCTGGGCTTCCAGTTCAC	CCTGTCTCGTTGCGTTTGTAGT
*Leptin*	Human	CGGAGAGTACAGTGAGCCA	CGGAATCTCGCTCTGTCAT
*Adiponectin*	Human	CCCAAAGAGGAGAGGAA	TCAGAAACAGGACACAAC

## Data Availability

The data presented in this study are available on request from the corresponding author.
